# A radiographic measurement of left atrial size in dogs

**DOI:** 10.1186/s13620-018-0137-x

**Published:** 2018-12-17

**Authors:** Xavier Sánchez Salguero, David Prandi, Francisco Llabrés-Díaz, Edgar Garcia Manzanilla, Claudio Bussadori

**Affiliations:** 1 Animal Science Department, Escola Tècnica Superior d’Enginyeria Agrària, Universitat de Lleida, Lleida, Spain; 2 Betulia Veterinary Clinic, Barcelona, Spain; 3 Universitat Autònoma de Barcelona, Bellaterra, Barcelona, Spain; 4 Royal Veterinary College, Hawkshead Lane, Hatfield, Hertfordshire UK; 5 Animal and Grassland Research Centre, Teagasc, Moorepark, Fermoy Republic of Ireland; 6 School of Veterinary Medicine, University College Dublin, Belfield, Republic of Ireland; 7 Clínica Veterinaria Gran Sasso, Milan, Italy

**Keywords:** Dog, Left atrial size, Mitral valve disease, Radiographic measurement

## Abstract

**Background:**

The dimensions of the left atrium in cases with mitral regurgitation are an indirect measurement of its severity. The objective of this study was to evaluate the value of a new radiographic measurement, the radiographic left atrial dimension (RLAD), for detecting left atrial enlargement (LAE) in dogs. Thirty one dogs without LAE and 46 dogs with LAE were recruited in a prospective fashion. Reference left atrium dimension was measured by standard left atrium to aorta ratio (LA/Ao) by 2D echocardiography. LAE was considered if LA/Ao > 1.6. Left atrium dimension was then quantified on lateral radiographs by measuring RLAD. Vertebral heart size (VHS) was measured and RLAD was obtained by drawing a line bisecting the 90 degrees angle defined by the long and short cardiac axes lines of the VHS, up to the dorsal edge of the left atrium and comparing its length to T4’s vertebral body length. The correlation of VHS and RLAD methods with LA/Ao was estimated, as well as their sensitivity and specificity for detecting LAE. Receiver Operating Characteristic (ROC) curves were used to estimate the optimal decision criteria for each method.

**Results:**

A positive correlation was observed between RLAD and LA/Ao (*r* = 0.82). RLAD’s sensitivity and specificity for detecting LAE when evaluated at the optimal cut-off value, 1.8 vertebrae, were 93.5 and 96.8% respectively. RLAD showed high reproducibility and repeatability.

**Conclusion:**

RLAD appears to be a clinically useful radiographic measurement for evaluating left atrial dimensions. RLAD would provide clinicians with a simple and cost-effective tool for evaluating and monitoring LAE.

## Background

Myxomatous mitral valve disease (MVD) is the most prevalent heart disease in dogs [1]. It is characterised by a progressive degeneration of the valvular apparatus leading to mitral regurgitation (MR) [1]. Thoracic radiographs are important in assessing the severity of mitral regurgitation (MR) caused by myxomatous mitral valve disease by determining the presence of generalised heart enlargement (HE) and left atrial enlargement (LAE) [1–3]. As the left atrium (LA) is the receiving chamber of the regurgitant volume, it is of great interest to have reliable methods available to quantify changes in LA dimensions. As the regurgitant fraction correlates to LA size it might be easier to monitor progression of MR by measuring changes of atrial dimension [4].

Echocardiography is the standard method for non-invasive assessment of cardiac dimensions, providing a direct and accurate means of estimating left atrial dimension. Several measurements have been described for this purpose and the most commonly used is the left atrium-to-aorta ratio (LA/Ao) derived from two-dimensional (2D) right parasternal short-axis ultrasonographic images at the level of the heart base [5–7]. Echocardiography is nonetheless technically challenging and not universally available. In the past, before the advent of echocardiography, evaluation of cardiac dimensions relied mostly upon radiographic assessment of the size and shape of the cardiac silhouette [8]. This widespread use has continued because it is available virtually to all veterinary clinicians and offers additional information such as the presence of pulmonary oedema. In general, however, its subjective and indirect character renders it prone to interpretation errors, even when used by experienced examiners [3, 8].

In 1995, Buchanan and Bücheler introduced the vertebral heart size (VHS), a standardised method of cardiac size evaluation using the thoracic vertebrae as a measurement unit [9]. This method is based on the good correlation existing between cardiac size and thoracic vertebral body’s length. To include the left atrial body, a slight variation was subsequently introduced, consisting in measuring the short axis from the dorsal edge of the caudal vena cava (CVC) [10]. The overall size of the heart is then normalized to body size by expressing it as units of vertebral length. This method provided an objective numerical measurement for evaluation of general heart size.

Thoracic radiographs are a sensitive means of evaluating LAE, especially when this is moderate to severe [8]. Several radiographic findings indicative of LAE have been described both in lateral and dorso-ventral views. The most commonly used is the observation of a dorso-caudally located bulge of soft tissue opacity, or left atrial “tent”, on the lateral radiograph [11, 12].

In this study, we sought to assess the usefulness of a new radiographic measurement (radiographic left atrial dimension, RLAD) for the evaluation of left atrial dimensions in normal dogs and dogs with MVD. The purpose of this study was three-fold: [1] to study the correlation between RLAD, VHS and LA/Ao, [2] to calculate sensitivity and specificity of VHS and RLAD to detect LAE using LA/Ao as a gold standard, and [3] to establish a reference value for RLAD in normal dogs. Intra- and inter-observer variability for VHS and RLAD were also determined.

## Methods

### Animals

A total of 77 dogs were prospectively included in this study. Fifty-four dogs were client-owned and were presented for a routine clinical examination and the remaining 23 belonged to a shelter facility. Dogs were assigned to one of two groups according to the absence (Group 1) or presence (Group 2) of LAE defined as LA/Ao > 1.6 [7]. Group 1 included 31 dogs, 22 males and 9 females, with a mean age of 4.2 years (1–7 years) and a mean weight of 15.8 kg (4.0–20.0 kg). Breeds represented in this group were mixed-breed (*n* = 25), Cocker Spaniel (n = 2) and one of each of the following: French Bulldog, Pug, Cavalier King Charles Spaniel and Pekingese. Group 2 included 46 mixed-breed dogs, 28 males and 18 females, with a mean age of 8.1 years (6–10 years) and a mean weight of 12.3 kg (4.8–21.4 kg).

All dogs underwent a complete clinical evaluation including physical examination, complete blood count, biochemistry panel, and radiographic and echocardiographic examinations within the same day. All dogs were examined by the same author in their respective centers of origin. Normal dogs were those considered so according to the history, physical, radiographic and echocardiographic examinations. A MVD diagnosis was based on the presence of a typical systolic mitral regurgitation murmur and alterations on the echocardiographic examination. Dogs presenting with radiographic and/or echocardiographic changes compatible with alveolar pulmonary oedema, pulmonary hypertension or right-sided cardiac disease, as well as cardiac diseases other than MVD, such as cardiomyopathy, myocarditis, congenital heart defects, and diagnosed arrhythmias were not included.

### Radiographic measurements

**Fig. 1:**
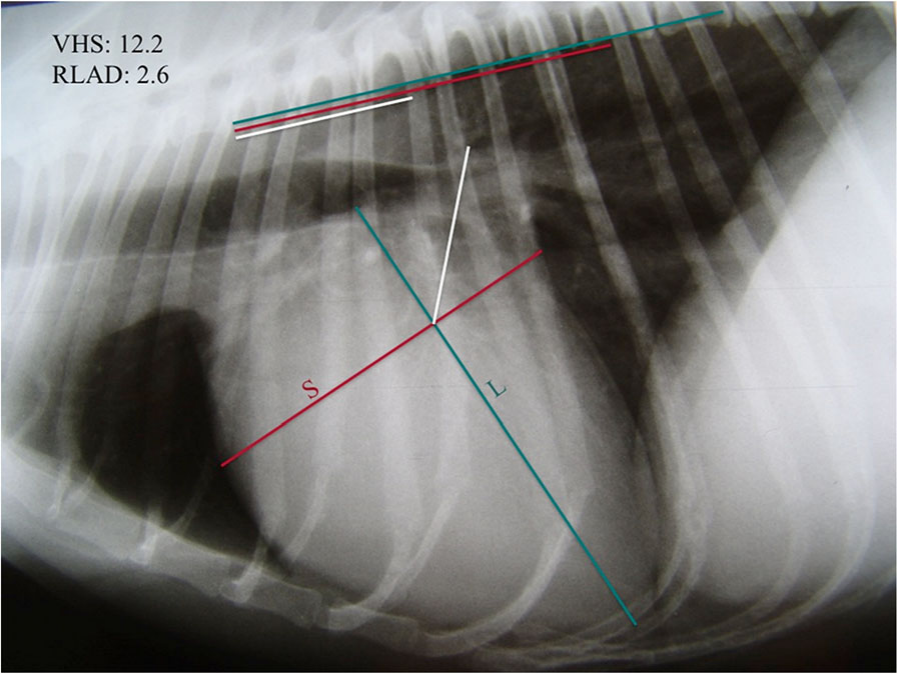
Right lateral thoracic view. Vertebral heart size (VHS), long (L) and short (S) cardiac axes are shown. VHS is expressed as total units of vertebral length: 12.2v. Radiographic left atrial dimension (RLAD) is shown (white line). Both measurements were repositioned parallel over thoracic vertebrae beginning with the cranial edge of the fourth thoracic vertebra (T4). The size of the LA was thus expressed as total units of vertebral length: 2.6v

A digital right lateral thoracic radiograph was used for measuring the VHS in each case, as described by Buchanan [10]. This procedure was performed by the same observer (XS) in all dogs. The long axis (L) was measured from the ventral border of the left main stem bronchus (carina) to the more distant point of the cardiac apex. The short axis (S) was measured starting at the level of the dorsal edge of the CVC (Fig. 1).

A commercially available computer software (Microsoft Office Power point 2001, Microsoft corporation, US) was used to apply a 90-degree rotation between L and S. L and S were then repositioned over the thoracic vertebrae from the cranial edge of the fourth thoracic vertebra, parallel to the vertebral column, and each length was then expressed in terms of the number of thoracic vertebrae (v) to the nearest 0.1v. The sum of the two was used as VHS (Fig. 1).

RLAD was obtained from the same radiographic images used for VHS quantification, and using the same computer software. This procedure was performed by the same observer (XS) in all dogs. A line bisecting the 90° angle formed by the intersection of the VHS L and S axes was drawn from this point to the radiographic projection of the dorsal edge of the left atrium (Fig. 1). The computer software was used to ensure a 45° angle between this line and the intersection of L and S. This length was then normalized to v starting at the cranial edge of the fourth thoracic vertebra and to the nearest 0.1v as for VHS and used as RLAD. In cases where it was difficult to differentiate the dorsal anatomical boundaries of the left atrium and the neighbouring pulmonary veins, the most dorsal aspect of the soft tissue opacity seen at this level was routinely used for all measurements.

### Echocardiographic measurements

A complete transthoracic echocardiographic examination (TTE) was performed in all dogs by the same clinician. The dogs were placed in right and left lateral recumbency and the examination was performed according to the American Society of Echocardiography standards and guidelines and other published recommendations [13].

Diagnosis of MVD was based on characteristic valvular lesions of the mitral valve apparatus (mitral valve thickening, prolapse or both) on 2D echocardiography and the identification of mitral valve regurgitation by colour Doppler examination, as described previously [14, 15]. The same criterion (LA/Ao value of 1.6) was used to define LAE [7].

**Fig. 2:**
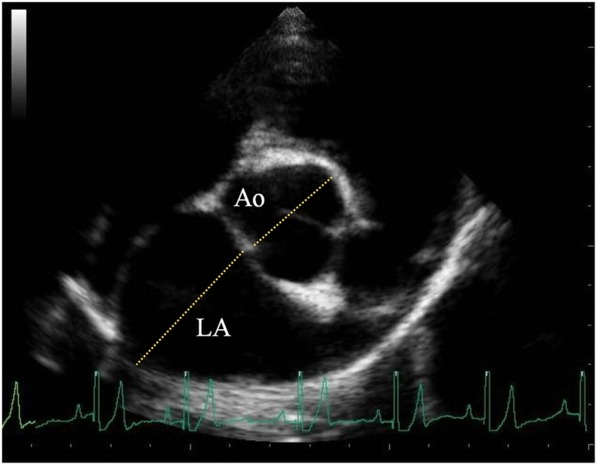
2D-right parasternal short-axis view at the aortic root level. The right coronary, left coronary and non-coronary cusps of the aorta may be seen. Aorta (Ao), left atrium (LA)

LA/Ao was obtained by calculating the ratio between the LA and cross-sectional aortic (Ao) diameters obtained by 2D TTE from a right parasternal short axis view as described in earlier reports [16–18]. The measurement was performed in early ventricular diastole using the first frame after aortic ejection where the Ao appeared as a symmetrical three-leaf clover with closed aortic valves and a teardrop-shaped LA (Fig. 2). Three measurements were performed in consecutive heart beats and averaged to reduce measurement error.

### Intra- and inter-observer variability for VHS and RLAD

Intra- and inter- observer agreement was studied using a complementary pilot study in which 2 different observers (DP and XS) measured VHS and RLAD in radiographs from 5 dogs from group 1 and 5 dogs from group 1. Each set of masked and randomized radiographs was evaluated in three occasions. Masking and randomization methods were applied in order to minimize potential biases. Observer bias was measured as the mean difference for all measurements between observers. Inter-observer variability was measured as the variability of the mean difference for each animal. Intra-observer variability was measured as the mean variability between replications for the same animal.

### Statistical analysis

Statistical analysis was performed using SAS 9.4 (Cary, NC, USA) and R software (Vienna, Austria). Alpha level for determination of significance was 0.05. Summary descriptive indexes (means, standard deviation and the corresponding minimum and maximum values) were obtained for the primary variables (VHS, RLAD, LA/Ao) in each subgroup. The association between measurements was obtained by means of Spearman’s correlation. The accuracy of each test was measured by means of empirical Receiver Operating Characteristic (ROC) curve, the area under the curve (AUC) and their confidence intervals. AUC curves were compare using Delong’s method [19]. The optimal cutoff value that jointly maximized sensitivity and specificity for each test was determined using the Younden index. Sensitivity and specificity and their confidence intervals were computed at the optimal cut-off point.

## Results

### Descriptive statistics

In group 1, 3 dogs presented with MVD and the remaining 28 were normal. In group 2, 44 dogs presented with MVD and 2 did not present with signs of valvular disease. Dogs with MVD were classified according to the ACVIM consensus panel guidelines for disease staging [20] as follows: 4 dogs in stage B1, 15 dogs in stage B2 and 28 dogs in stage C.

Dogs in stage A were patients at high risk for developing heart disease but that currently had no identifiable structural disorder of the heart. Dogs in stage B were patients with structural heart disease but that had not developed clinical signs associated to heart failure (Stage B1: asymptomatic patients that had no radiographic or echocardiographic evidence of cardiac remodeling. Stage B2: asymptomatic patients that had hemodynamically significant regurgitation, as evidenced by radiographic or echocardiographic findings of left-sided heart enlargement). Dogs in stage C were patients with past or current clinical signs of heart failure associated with structural heart disease. Dogs in stage D were patients with end-stage disease with clinical signs of heart failure that were refractory to standard therapy.

**Fig. 3:**
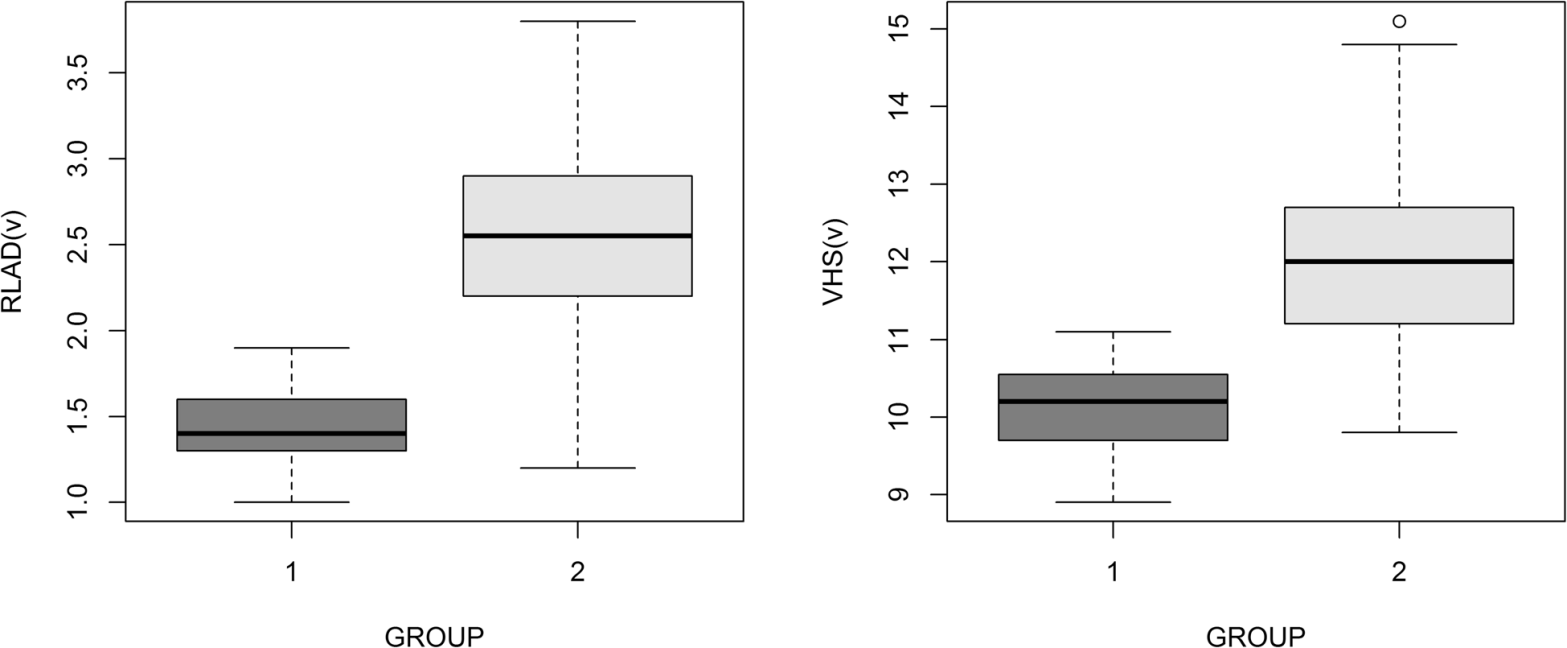
Boxplots comparing radiographic left atrial dimension (RLAD) and vertebral heart size (VHS) measures for groups with absence (Group 1, *n* = 31) or presence (Group 2, *n* = 46) of left atrial enlargement defined as La/Ao > 1.6. The box shows the median and quartiles and the whiskers represent the range or 1.5 times inter-quartile range in the presence of outliers

Group 2 showed greater values for all variables (*P* < 0.05). Mean (± SD) RLAD value was 1.41 ± 0.23 in group 1, and 2.54 ± 0.52 in group 2; mean VHS value was 10.19 ± 0.60 in group 1, and 12.05 ± 1.17 in group 2 and finally, mean LA/Ao value was 1.33 ± 0.13 in group 1, and 2.47 ± 0.55 in group 2. Box plots for RLAD and VHS are shown in Fig. 3.

### Spearman’s correlation, sensitivity and specificity

A positive correlation (*r* = 0.79, *P* < 0.001) was observed between VHS and RLAD, between VHS and LA/Ao (*r* = 0.82, *P* < 0.001) and between RLAD and LA/Ao (*r* = 0.84, *P* < 0.001).

**Fig. 4:**
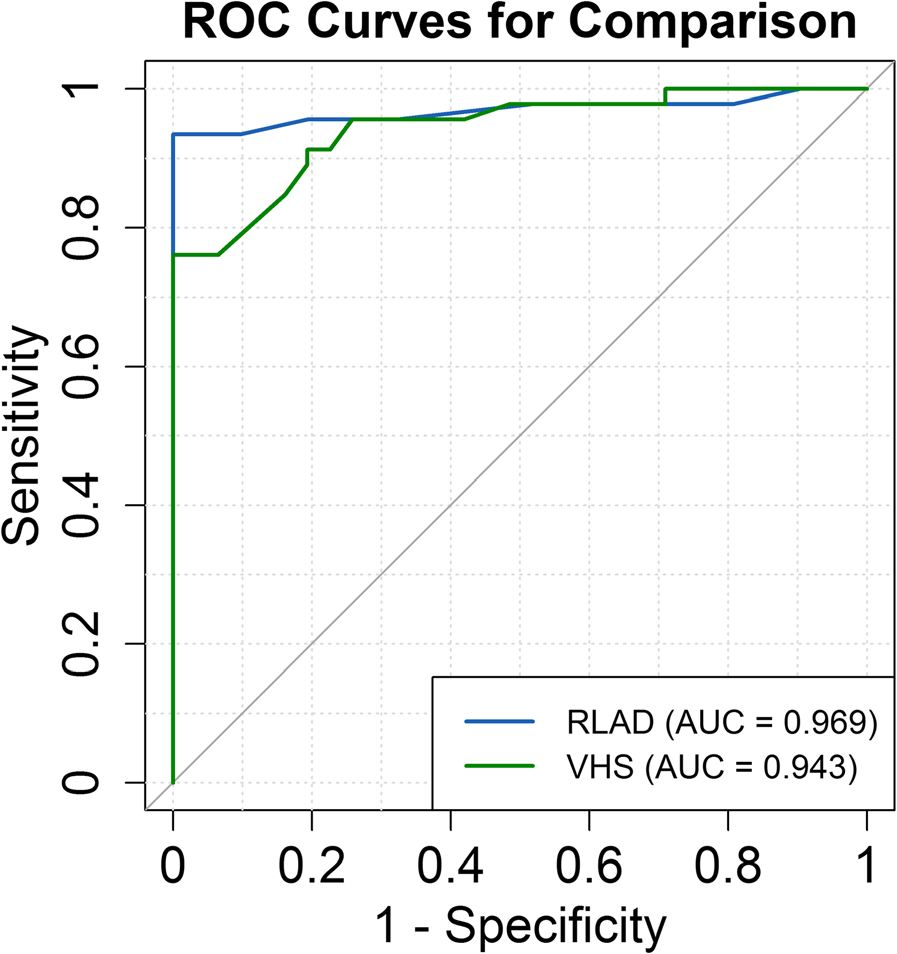
Comparisons between ROC curves and areas under the curve (AUC) for variables radiographic left atrial dimension (RLAD) and vertebral heart size (VHS). The optimal cut-off point (reference value) for these variables are shown and quantified in 1.8 and 11.1 thoracic vertebrae. AUC for RLAD was 0.97 and AUC for VHS was 0.94

Sensitivity and specificity values determined for RLAD and VHS were considered regarding the detection of LAE with the criterion LA/Ao > 1.6. The sensitivity and specificity for RLAD were 93.5% (84.78, 100) and 96.8% (90.32, 100); whereas for VHS these indices were 76.1% (63.04, 87.01) and 93.5% (83.87, 100). ROC curve and AUC for RLAD and VHS are depicted in Fig. 4. No statistical differences between AUC values were present, *P* = 0.274 (− 0.021–0.074). The RLAD and VHS optimal cut-off values which provided the greatest sensitivity and specificity for detecting LAE (maximal reference values) were of 1.8v and 11.1v respectively.

**Fig. 5:**
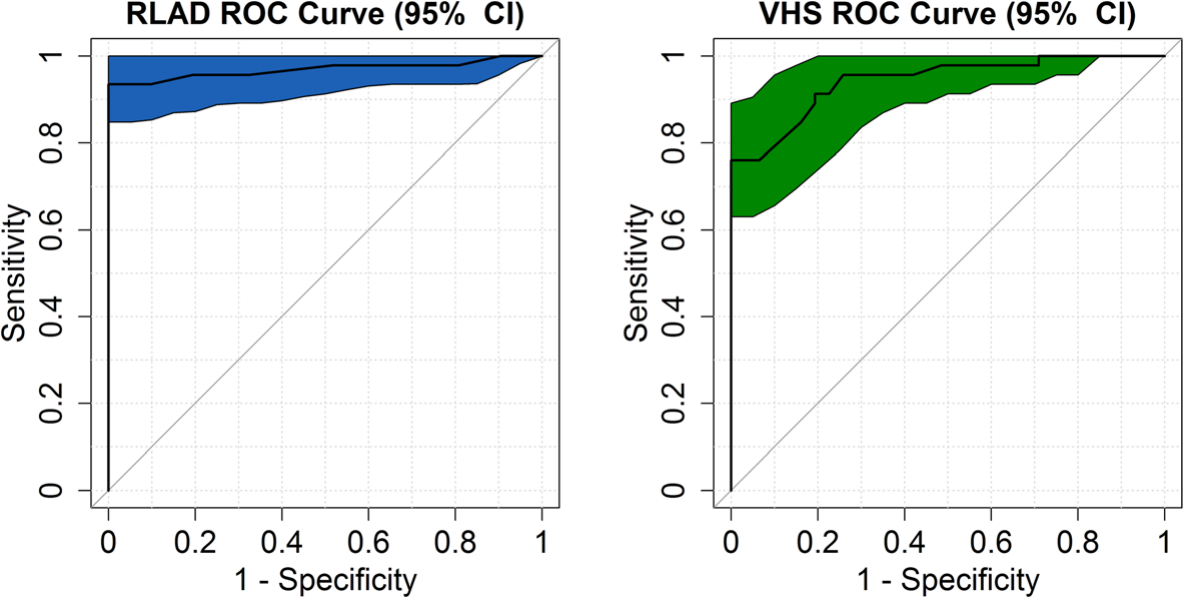
95% confidence intervals for ROC curves of variables radiographic left atrial dimension (RLAD) and vertebral heart size (VHS)

The AUC and Confidence limits for RLAD and VHS were respectively 0.9691 (0.9281–1.00) and 0.9425 (0.8952, 0.9898). The confidence interval of ROC curves for RLAD and VHS are depicted in Fig. 5.

### Intra- and inter-observer variability

Regarding the VHS, observer 1 (DP) demonstrated a 0.08v variability between measurements whereas observer 2’s (XS) was 0.03v (Table 1). Regarding RLAD, both observers demonstrated a 0.01v variability between measurements. An inter-observer variability of 0.26v and 0.17v was found for VHS and RLAD measurements with observer biases of − 0.09 and 0.06 respectively.

**Table 1:** Intra-observer agreement for VHS and RLAD

	Observer	N	Mean	SD	CV	Intra-Obs Var
VHS	1	30	11.36v	0.48v	4.22	0.08v
	2	30	11.44v	0.59v	5.16	0.01v
RLAD	1	30	1.97v	0.57v	29.03	0.03v
	2	30	1.91v	0.63v	32.67	0.01v

*N* Number of observations, *SD* Standard Deviation, *CV* Coefficient of Variation, Intra-*Obs Var* Intra-Observer variability, *v* thoracic vertebrae

## Discussion

This study describes a novel objective measurement (RLAD) to estimate LAE in dogs using standard thoracic radiographs. The main goal of this study is to describe and evaluate a new radiographic measurement to detect increases of left atrial size in dogs. Cardiac radiology has been shown to be especially reliable to show increases of the left atrium size [1–3] and to date, there is no objective radiographic measurement (such as VHS to detect cardiomegaly) to detect and quantify these increases in left atrial size. LA/Ao ratio is the most commonly used echocardiographic measurement to detect LAE [5–7]. Using an index (LA/Ao ratio) to determine LAE is superior to using other LA measurements normalized to body weight or body surface area, as the index is an independent internal ratio [6]. The RLAD was devised to measure the extent of this “bulging” through a VHS-derived measurement and therefore estimate left atrial dimensions. In cases with only mild LAE, it can be difficult to accurately differentiate the dorsal edge of the left atrium due to the superimposition of the bronchial tree but also and most importantly, the neighbouring pulmonary veins. The latter will demonstrate the same radiographic opacity. Previous radiographic references have almost certainly included these veins within the soft tissue opacity described as LAE [11]. This difficulty in differentiating the boundaries of the left atrium from the neighbouring pulmonary veins was encountered in only one patient in this study. This is, nonetheless, a potential pitfall of the RLAD in cases with mild LAE. Therefore, the authors recommend including the most dorsal aspect of the soft tissue opacity situated caudal to the carina for consistency of the obtained measurements. The dorsal edge of a moderate or severe LAE is easy to differentiate from the veins.

The usefulness of RLAD is shown by its strong correlation (*r* = 0.84) with LA/Ao in this study. Similarly, a good correlation was also observed between VHS and LA/Ao (*r* = 0.80), although not as strong as in experimentally-induced cardiomegaly (*r* = 0.9) [21]. Therefore, RLAD and VHS provide therefore a good estimate of left atrial dimensions when compared to the current standard method used for this purpose (LA/Ao).

In order to assess the value of these two measurements for the quantification of LAE, their respective sensitivity and specificity for this purpose was estimated and optimal cut-off values were determined. In our study, RLAD proved to be a more sensitive (93.5%) and specific (96.8%) tool for this purpose, with an optimal cut-off value of 1.8v. VHS was less sensitive (76.1%) and specific (93.5%) with an optimal cut-off value of 11.1v. In any case, both VHS and RLAD would be available for patients examined because VHS needs to be performed to have the center-point for RLAD. The optimal cut-off values obtained in this study for VHS were higher than those reported previously [9, 10]. This may be due to 1) differences in study populations, especially regarding the breeds included and different patterns of LA enlargement in different stages of disease, 2) inter-observer variability [22] and 3) the authors defined normal as LA/Ao ≤ 1.6.

Intra- and inter-observer variability were also assessed for RLAD to determine its repeatability and reproducibility, both important characteristics for any objective measurement tool, and since one landmark for RLAD is the bisection of the VHS axes, the same was performed for the latter. No significant differences were found in the level of agreement between observers for both indexes VHS and RLAD. Mean and CV for both radiographic variables were similar between observers. Intra-observer variability was small, especially in the case of RLAD, with intra-observer variability being 0.01, confirming the repeatability of the measurements for the same observer. Observer bias was also negligible for both tests. Thus, a high reproducibility and repeatability were observed for RLAD and VHS.

This study has some limitations. The MVD population in this study consisted of a series of dogs arbitrarily presented to cardiology referral centres. In this way, this population may not be representative of the general canine population with MVD, possibly including a higher proportion of dogs in a more advanced disease state which would increase the sensitivity of the test. On the other hand, given the large number of cross breeds included, a breed-dependent effect could not be evaluated in this study but would be of interest for future studies.

In fact, 24 out of 46 dogs in group 2 were classified in disease stage C, with 37 out of 46 dogs presenting with a LA/Ao > 2. This fact contributes to the high correlation observed between RLAD and LA/Ao. Further studies are needed in order to obtain clinically useful cut-off values for mild, moderate and severe left atrial enlargement, especially in dogs presenting with only mild to moderate LAE.

Furthermore LAE was estimated only by 2D echocardiography, which although widely accepted as a non invasive clinically useful method, it does not constitute a real gold standard method for this purpose as could be MRI (magnetic resonance imaging) or CT. Cross-sectional imaging would constitute a useful comparison in future studies involving LAE assessment with RLAD as a gold standard method.

## Conclusion

The new radiographic measurement named RLAD demonstrated high sensitivity and specificity for detecting LAE with a strong correlation with LA/Ao ratio. The proposed optimal cut-off value for RLAD to detect LAE is 1.8v. RLAD would provide clinicians with a simple and cost-effective tool for the detection and monitoring of LAE in dogs with MVD and possibly dogs with other cardiac diseases presenting with LAE.
